# To prescreen or not to prescreen for broadly neutralizing antibody sensitivity in HIV cure-related trials

**DOI:** 10.1016/j.jve.2023.100339

**Published:** 2023-07-18

**Authors:** Hursch Patel, Karine Dubé

**Affiliations:** a University of California San Diego School of Medicine, Division of Infectious Diseases and Global Public Health (IDGPH), La Jolla, San Diego, CA, USA; b University of North Carolina Gillings School of Global Public Health, Chapel Hill, NC, USA

**Keywords:** Broadly neutralizing antibodies, Pre-screening, Sensitivity, HIV cure research

## Abstract

The use of broadly neutralizing antibodies (bNAbs) as a cure-related research strategy for human immunodeficiency virus (HIV) has gained attention from the scientific community. bNAbs are specialized antibodies that target HIV-1 by binding to proteins on the surface of the virus, preventing the infection of human cells. In HIV-1 clinical studies assessing the use of bNAbs, it has been common practice to prescreen potential participants for bNAb sensitivity. However, the use of pre-screening in HIV-1 bNAb clinical trials is a topic of ongoing debate, with regard to its potential benefits and limitations. In this paper, we examine the possible benefits and limitations of pre-screening for bNAb sensitivity in HIV-1 cure-related studies, and suggest alternative methods which may be more effective or efficient at saving costs and time. Ultimately, the decision to use pre-screening in HIV-1 bNAb clinical trials should be based on a careful assessment of the potential benefits and limitations of this approach, as well as the specific needs, goals, design, and population of the study in question.

## 1 Introduction

Human immunodeficiency virus type-1 (HIV-1) is an ongoing global health issue that has claimed the lives of millions of people worldwide.^1^ While antiretroviral treatment (ART) has significantly reduced morbidity and mortality associated with HIV-1, it does not eradicate the virus.^2^, ^3^, ^4^ To this extent, the development of HIV-1 broadly neutralizing antibodies (bNAbs) is of growing interest to the HIV-1 cure scientific community.^5^ BNAbs are specialized antibodies that can target HIV-1 directly by binding to proteins on the surface of the virus to prevent the infection of human cells.^6^ Consequently, bNAbs can neutralize a wide range of HIV-1 strains, and combinations have been found to broaden antiviral coverage.^6^ With the promising advantages bNAbs have shown in helping control HIV-1 combined with enhancements – such as increased breadth, potency, broader, extended bioavailability, novel delivery platforms, and combinations with other agents – clinical studies assessing their use as a potential treatment option are growing.^6^, ^7^, ^9^, ^8^ In these studies, it has been common practice to prescreen potential participants for bNAb sensitivity to ensure that they are appropriate trial candidates. Pre-screening involves identifying individuals who are likely to display viral sensitivity to the bNAb(s) under investigation, using a pre-defined assay, prior to trial enrollment.^10^ Pre-screening is an important study consideration as it has been well-documented that those individuals with resistance are less likely to respond to the bNAb intervention and achieve sustained ART-free viral control.^11^ Such information should therefore be included in informed consent forms for any future study that forgoes pre-screening. However, there is an ongoing debate regarding the effectiveness and value of pre-screening in bNAb clinical trials, as well as the accuracy of the pre-screening methods that are currently available.

In this viewpoint paper, we examine the benefits and limitations of pre-screening in HIV-1 bNAb studies. We then outline possible avenues for future research.

## 2 Discussion

### 2.1 Pre-screening information

There are currently four main methods used in the pre-screening of HIV-1 bNAb clinical trials: the PhenoSense assay, the TZM-bl neutralization assay, Genotyping, and the Sequencing Q4 assay.

### 2.2 Arguments for prescreening for bNAb sensitivity

Many clinical trials have integrated pre-screening into their study protocols, taking HIV-1 isolates from people with HIV (PWH) and testing these samples against bNAbs to determine if these trial candidates will respond to the bNAb intervention.^10^^,^^14^, ^15^, ^16^, ^17^, ^18^, ^19^, ^20^, ^21^ Pre-screening supports the idea that trial enrollment should be limited to those most likely to benefit from the bNAb in question. This technique reduces the number of participants needed to demonstrate the initial effectiveness of a bNAb.^22^ Consequently, one of the main reasons for pre-screening is to not only ensure that the study sample is representative of the population of interest, but also to reduce the risk of adverse events and protect individuals who may not benefit from treatment from undergoing unnecessary risks. Through prescreening, researchers can increase the validity and reliability of their results around bNAb efficacy.

Another common reason for pre-screening involves the high diversity within the HIV-1 reservoir in addition to HIV-1 high mutation rates, both of which account for viral isolates frequently becoming resistant to certain bNAbs and can be detected by bNAb assays using DNA and RNA, respectively.^23^, ^24^, ^25^ As reviewed by Yu and colleagues, there have been many reports from both animal and clinical trials stating that bNAb treatment failure has occurred due to the rapid selection of bNAb-resistant viral variants.^23^ Additionally, in a separate study assessing the bNAbs 3BNC117 and 10–1074, Bar-On et al. have found that only 67% and 58% of trial candidates tested showed sensitivity to 3BNC117 and 10–1074 on prescreening data from a TZM-bl assay, respectively.^26^ Other researchers like Wilson et al. have explicitly stated that pre-screening participants for neutralization sensitivity directly increased the effectiveness of the bNAbs under investigation, emphasizing the importance of developing future methods to overcome bNAb resistance.^27^^,^^28^

Additionally, pre-screening can potentially reduce the overall cost and time required for studies by streamlining the recruitment process and minimizing the risk of enrolling individuals who may not be eligible or suitable for the study. However, this argument is controversial given the cost of pre-screening and other disadvantages addressed in Table 1.

**Table 1:** Comparison of the PhenoSense assay, TZM-bl neutralization assay, genotyping, and sequencing Q4 assay for pre-screening in HIV broadly neutralising antibody (bNAb) clinical trials.

Method	Description	Logistics	Advantages	Disadvantages
PhenoSense Monogram assay	Measures the response of an individual's virus to antiretroviral therapy (ART)	Cost: Hight ime: several days to weeks Effort: specialized equipment and personnel training	Provides insights regarding an individual's susceptibility to various antiretroviral therapies	High cost and time
TZM-bl Neutralization assay	Measures the neutralization capacity of antibody against various strains of HIV-1	Cost: hight ime: several days Effort: specialized equipment and personnel training	Provides a functional measure of the neutralizing capacity of antibodies	High cost and time
Genotyping	Analyzes the genetic composition of HIV-1 to predict antibody sensitivity	Cost: lowt ime: several days Effort: computational database	Provides mutational sequencing information from the virus genetic composition	Limited by availability of sequencing datasets
Sequencing Q4 assay	Analyzes HIV-1 genetic composition at different timepoints to predict antibody sensitivity	Cost: lowt ime: several days Effort: computational database	Provides specific sequencing information as the virus evolves	Multiple samples required at different timepoints. Limited by availability of sequencing datasets

### 2.3 Arguments for not pre-screening for bNAb sensitivity

While pre-screening has been used in several bNAb clinical trials, it is not without its limitations. One main area of concern is the quality of the assays that are available. For instance, in a study by Mendoza et al., the TZM-bl neutralization assay failed to detect pre-existing resistance that was present in 2 out of 11 individuals, a pattern evidenced by several other studies within the current literature.^26^^,^^29^ A possible explanation for these findings is that the viral samples collected for the pre-screening assays may not be representative of an individual's entire and diverse HIV-1 reservoir.^26^^,^^29^ This limitation is exacerbated by the cost, time, and labor required to complete these assays, especially in studies with large numbers of potential participants.^30^^,^^31^

Another limitation is the finding that pre-screening results, which may indicate bNAb sensitivity, do not always correspond with actual bNAb sensitivity.^32^ As shown by Liu et al., bNAb trial participants were found to be highly sensitive to 3BNC117 based on pre-screening data, but did not achieve complete neutralization against HIV-1, even at high antibody concentrations, leading researchers to question the accuracy of pre-screening as a description of bNAb neutralizing sensitivity.^33^

Additionally, many HIV bNAb studies have been successfully conducted without the use of pre-screening.^8^^,^^30^^,^^34^^,^^35^ For instance, Gaebler et al. did not prescreen their participants and later found that 76% of them were able to maintain virological suppression for a period of time after bNAb treatment.^8^ In fact, Hraber et al. have retrospectively concluded that prescreening was not necessary due to the high frequency of bNAb sensitivity demonstrated in their randomly selected group which did not go through pre-screening.^36^ This group further suggested that pre-screening could bias datasets in favor of higher levels of neutralization activity.^36^

Other potential limitations of pre-screening for bNAb sensitivity involve accessibility considerations, such as the exclusion of certain groups or individuals from study participation. For example, some individuals may be excluded due to pre-existing conditions or other factors that could interfere with the study, which may raise concerns about access and equity. This can be seen in individuals with non-clade B virus who may be excluded from studies testing bNAbs which are optimized for clade B virus. Additionally, pre-screening may also raise concerns about the representativeness of the study sample, as certain groups or individuals may be disproportionately excluded due to pre-screening criteria. Furthermore, it is important to consider study designs as a limitation, particularly those involving newly-infected individuals with HIV-1 for whom pre-screening would inappropriately delay bNAb treatment.

### 2.4 Possible avenues for future research

Outside of the assay-based methods used for bNAb pre-screening, some researchers have recommended genotypic assessments as an alternative approach. Genotypic assessments analyze the nucleotide sequences of HIV-1 to identify mutations or variations which may impact on the susceptibility to certain bNAbs.^10^^,^^37^ This technique is more efficient and cost-effective in comparison to assay-based methods and has been used by numerous HIV-1 bNAb studies.^10^^,^^38^, ^39^, ^40^, ^41^, ^42^, ^43^, ^44^, ^45^, ^46^, ^47^, ^48^, ^49^, ^50^, ^51^ Of note, Gaebler et al. have found that, in comparison to neutralization assays, genotypic prediction to identify resistance was inadequate, suggesting further room for improvement.^8^

Another alternative to assay-based pre-screening is the use of phenotyping, which measures HIV-1 replication in the presence of different drugs.^53^ Studies using phenotyping have been able to accurately predict bNAb resistance of HIV-1 isolates in a less time-consuming and expensive way than the assay-based pre-screens.^24^ Furthermore, Moldt et al. have found that phenotyping was as predictive as genotyping when determining bNAb sensitivity.^10^ However, as Zacharopoulou et al. have stated, the main issue for computational techniques such as phenotyping is the limited availability of neutralization datasets.^52^

A growing number of HIV-1 bNAb clinical trials are replacing pre-screening with *post-hoc* analysis.^8^^,^^20^^,^^54^ By retrospectively examining data, researchers can save the time and money that would have otherwise been spent on pre-screening, while still having the option to identify factors associated with successful bNAb outcomes.

The field is currently in a state of equipoise regarding the use of pre-screening for HIV-1 bNAb clinical trials, questioning if pre-screening is a necessary step in study designs. Despite this many investigators using bNAbs are opting not to include pre-screening due to practical limitations – cost, time, and effort – as outlined in Supplementary Table 1 which compiles data on recent bNAb studies and their use or not of pre-screening. With the aforementioned alternatives to bNAb pre-screening neutralization assays, further research is needed to formulate the best approach to enrolling participants into bNAb clinical studies.

## 3 Conclusions

The use of pre-screening in HIV-1 bNAb clinical trials is undetermined, with an ongoing debate regarding its potential benefits and limitations. On one hand, pre-screening has the potential to increase the validity and reliability of a study by ensuring that the study sample is representative of the population being studied and by reducing the potential for confounding factors or biases. On the other hand, the accuracy and reliability of the assays used for pre-screening, the cost and time required to complete these assays, and considerations related to the exclusion of certain groups or individuals from participating in such studies, represent limitations. Overall, the use of pre-screening in HIV-1 bNAb clinical trials is a complex issue that requires careful evaluation. It is important to consider alternative methods, which may be more effective or efficient in reducing costs and time. Ultimately, the decision to use pre-screening should be based on a careful assessment of the potential benefits and limitations of this approach, as well as the specific needs, goals, design, and type of study population.

## Funding

K.D. has received support from UM1AI126620 (BEAT-HIV Collaboratory; PI: Luis Montaner) co-funded by 10.13039/100000060NIAID, 10.13039/100000025NIMH, 10.13039/100000065NINDS and 10.13039/100000026NIDA).

## Declaration of competing interest

The authors declare the following financial interests/personal relationships which may be considered as potential competing interests: KD serves as a consultant for Gilead Sciences, Inc.

## Data Availability

Data will be made available on request.
